# Supplementary material for: Structures of human organellar SPFH protein complexes
Source: Nat Commun. 2025 Nov 17;16:10064. doi: 10.1038/s41467-025-65078-3 (PMC12624060; doi:10.1038/s41467-025-65078-3)
Supplement: Supplementary file 1 — Supplementary Information [file 41467_2025_65078_MOESM1_ESM.pdf]

Structures of human organellar SPFH protein complexes

Jingjing Gao, Dawafuti Sherpa, Nikita Kupko, Haruka Chino, Jianwei Zeng, Sichen Shao

Supplementary Information

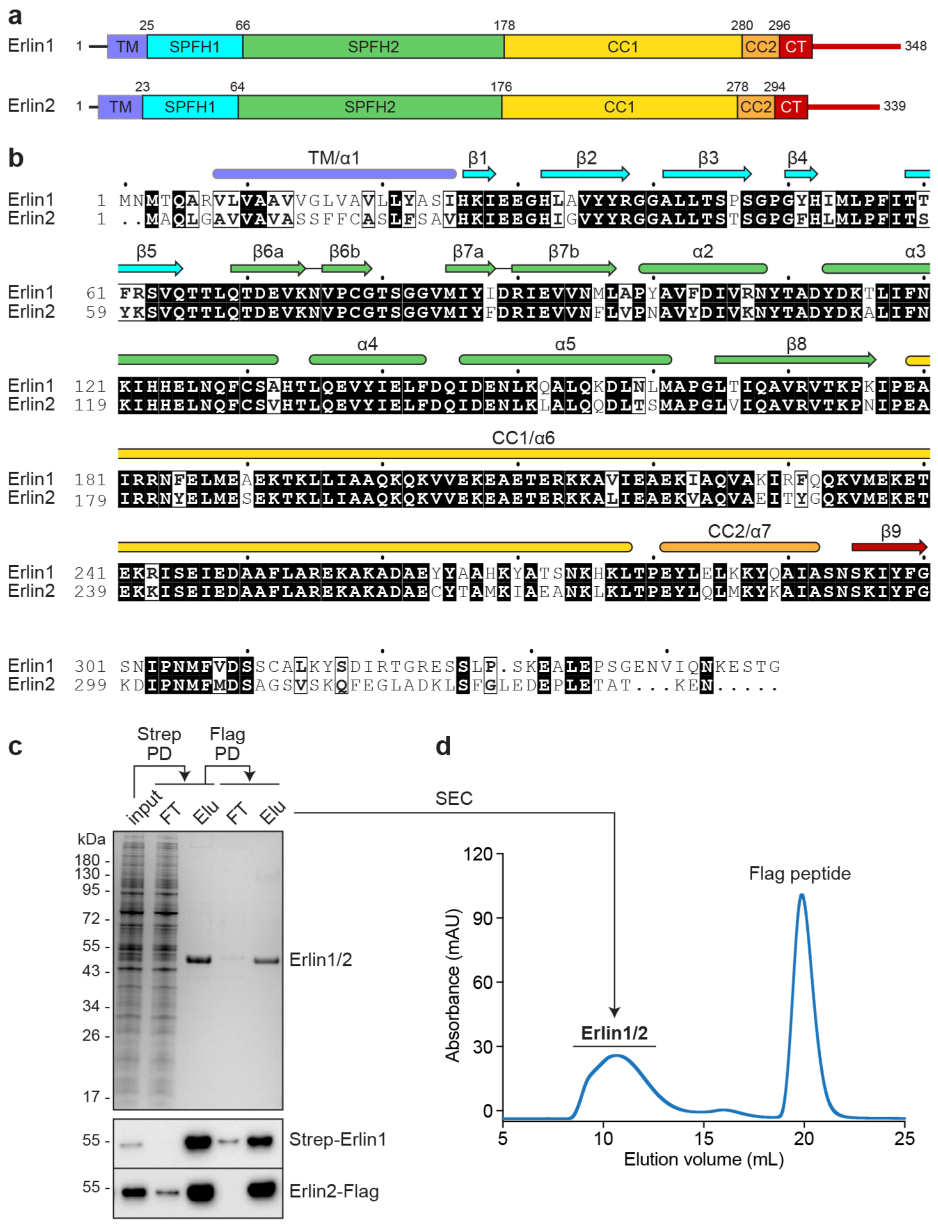

Supplementary Fig. 1. Alignment and purification of Erlin1 and Erlin2. a, Domain scheme of Erlin1 and Erlin2. **b,** Sequence alignment of human Erlin1 and Erlin2, with structural features noted above, colored according to domain. **c,** Coomassie stain (top) and immunoblotting (bottom) of the Erlin1/2 complex tandemly purified via N-terminally Strep-tagged Erlin1 and C-terminally Flag-tagged Erlin2. PD, pulldown; FT, flow-through; Elu, elution. **d,** Size exclusion chromatography (SEC) trace, showing absorbance at 280 nm values, of the tandem-purified Erlin1/2 complex as in c. The fractions corresponding to Erlin1/2 as indicated were pooled for cryo-EM analysis. Source data are provided as a Source Data file.

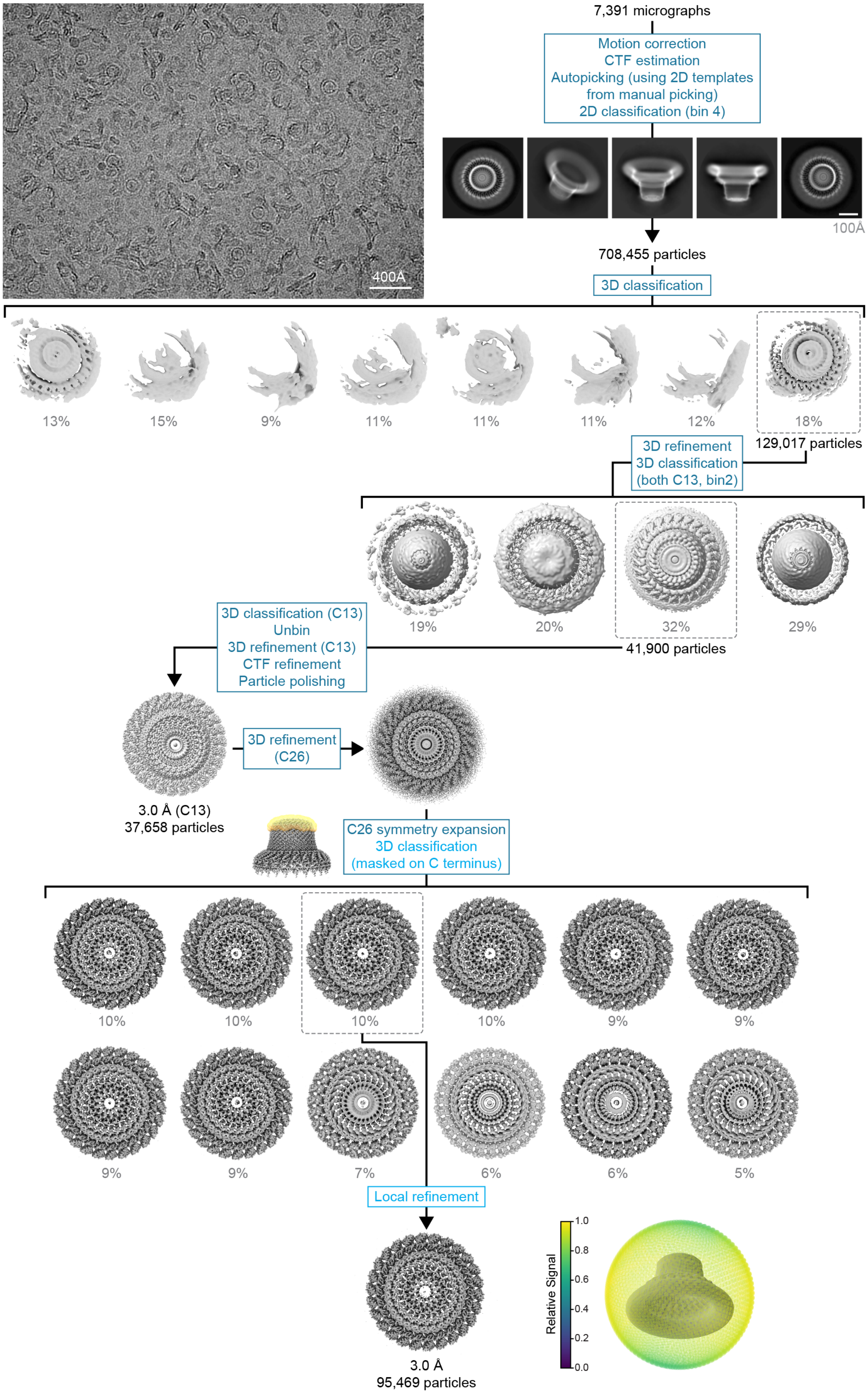

400Å
7,391 micrographs
Motion correction
CTF estimation
Autopicking (using 2D templates from manual picking)
2D classification (bin 4)
100Å
708,455 particles
3D classification
13%
15%
9%
11%
11%
11%
12%
18%
129,017 particles
3D refinement
3D classification
(both C13, bin2)
19%
20%
32%
29%
41,900 particles
3D classification (C13)
Unbin
3D refinement (C13)
CTF refinement
Particle polishing
3.0 Å (C13)
37,658 particles
3D refinement
(C26)
C26 symmetry expansion
3D classification
(masked on C terminus)
10%
10%
10%
10%
9%
9%
9%
9%
7%
6%
6%
5%
Local refinement
3.0 Å
95,469 particles
Relative Signal
1.0
0.8
0.6
0.4
0.2
0.0

Supplementary Fig. 2. Cryo-EM data processing pipeline for the Erlin1/2 complex. Representative micrograph (top left) and cryo-EM processing pipeline for the Erlin1/2 complex, showing representative 2D classes, 3D volumes, and the angular distribution of particles in the final reconstruction (bottom right). Processing steps performed using RELION are in teal; processing steps performed using cryoSPARC are in light blue.

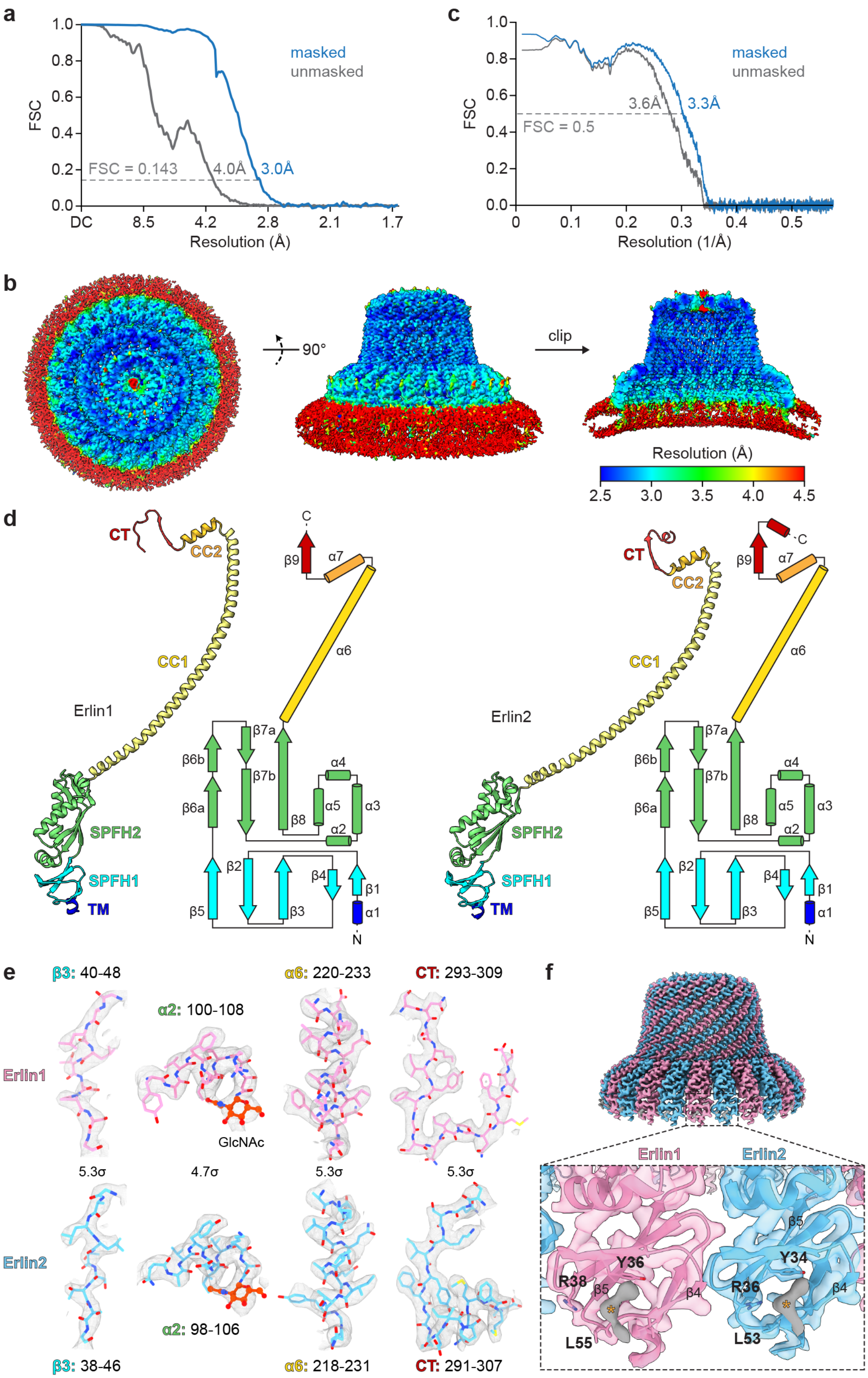

Supplementary Fig. 3. Quality of the Erlin1/2 complex map and model. a, Fourier shell correlation (FSC) vs. resolution curves for the cryo-EM map of the Erlin1/2 complex. **b,** Top

(left), side (middle), and clipped (right) views of the Erlin1/2 complex map colored by local resolution. **c,** Model vs. map FSC curves. **d,** Model and secondary structural element organization of Erlin1 (left) and Erlin2 (right), colored by domain. **e,** Map-to-model fits for the indicated regions of Erlin1 (top) or Erlin2 (bottom). **f,** Cryo-EM map of the Erlin1/2 complex with inset showing additional unidentified density (gray, orange asterisks) associated with the SPFH1 domain of each subunit. Source data are provided as a Source Data file.

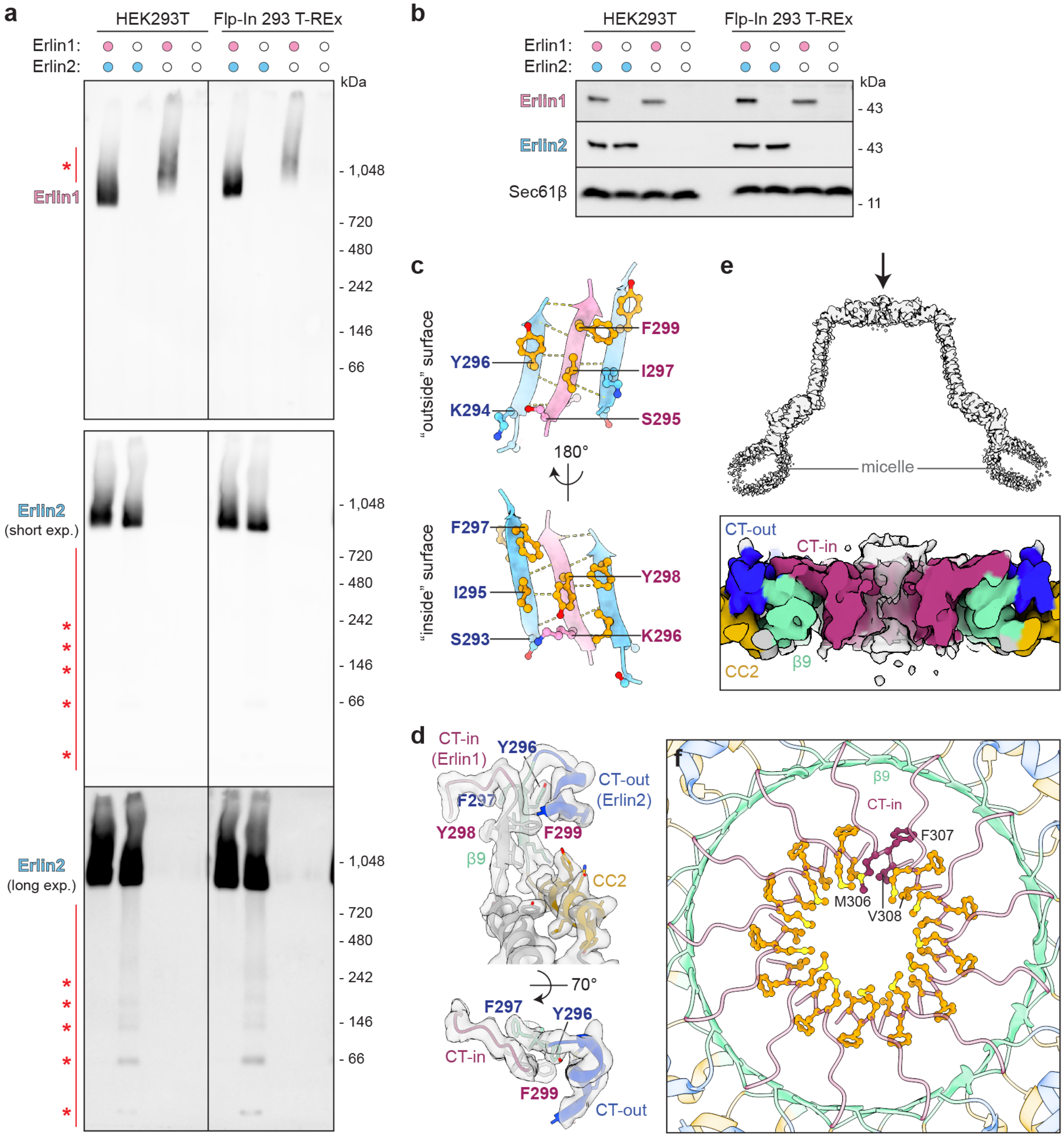

Supplementary Fig. 4. Organization of the Erlin1/2 complex. a, BN-PAGE and immunoblotting for Erlin1 and Erlin2 in HEK293T or Flp-In 293 T-Rex cells without (closed circles) or with (open circles) Erlin1 and/or Erlin2 knocked out. Red asterisks and lines indicate changes in Erlin1 or Erlin2 migration upon deletion of the other paralog. Note: Erlin1 assemblies increase in size in the absence of Erlin2 while Erlin2 is more likely to be found in lower molecular weight assemblies in the absence of Erlin1. **b,** SDS-PAGE and immunoblotting of samples from a. **c,** Sidechains of the residues in parallel β9 strands, viewed from either the 'outside' or the center ('inside') of the Erlin1/2 complex cage. Note: the β9 sequences are identical, but the side that each sidechain resides alternates between adjacent Erlin subunits. **d,** Map-to-model fit of the C-terminal Erlin elements, viewed from the outside (top) or top (bottom) of the Erlin1/2 complex cage, with aromatic residues in β9, as in c, indicated. Note: clear divergence in the position of alternating CTs. **e,** Side view of the cryo-EM map of the Erlin1/2

complex, clipped near the center (top), shows a potential central channel (arrow) flanked by CT-in elements (inset at bottom). **f,** Top view of the Erlin1/2 complex model showing hydrophobic residues in the CT-in element (modeled as Erlin1) that flank a central channel-like structure. Source data are provided as a Source Data file.

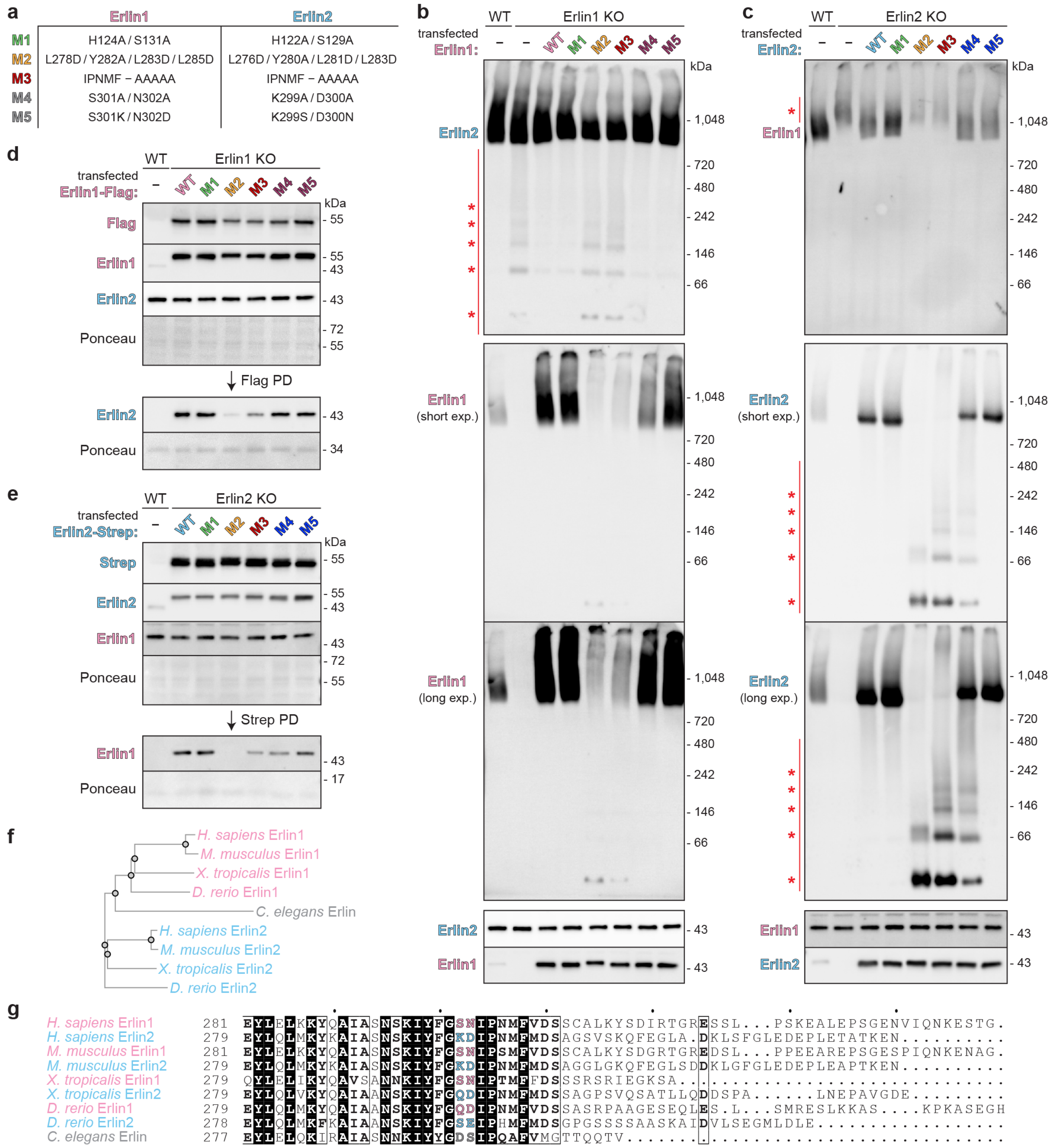

Supplementary Fig. 5. Impacts of Erlin mutations on complex assembly. a, Table showing the mutations present in the Erlin1 and Erlin2 variants tested. **b,c,** BN-PAGE (top) or SDS-PAGE (bottom) and immunoblotting of wildtype (WT) HEK293T cells and either **b,** Erlin1 knockout (KO) cells re-expressing the indicated Erlin1 variants or **c,** Erlin2 KO cells re-expressing the indicated Erlin2 variants. Note: re-expression of the WT version of each Erlin paralog rescues the defective assembly signatures (red asterisks and lines) of the other Erlin paralog. **d,e,** SDS-PAGE and immunoblotting of the input (top) and the indicated pulldowns (PD) of lysates from WT HEK293T cells and from **d,** Erlin1 KO cells re-expressing the indicated C-terminally Flag-tagged Erlin1 variants, or **e,** Erlin2 KO cells re-expressing the indicated C-

terminally Strep-tagged Erlin2 variants. Note: the M2 and M3 variants show defects in pulling down the other Erlin paralog. **f,** Phylogenetic tree via neighbor joining, of the Erlin paralog sequences from the indicated organisms. **g,** Sequence alignment of the C-terminal regions of Erlin paralogs from the indicated organism, with the paralog-distinct residues following β9 highlighted. Note: the polar nature, but not the exact identities, of these amino acids are conserved in Erlin1 versus Erlin2 paralogs across species. Source data are provided as a Source Data file.

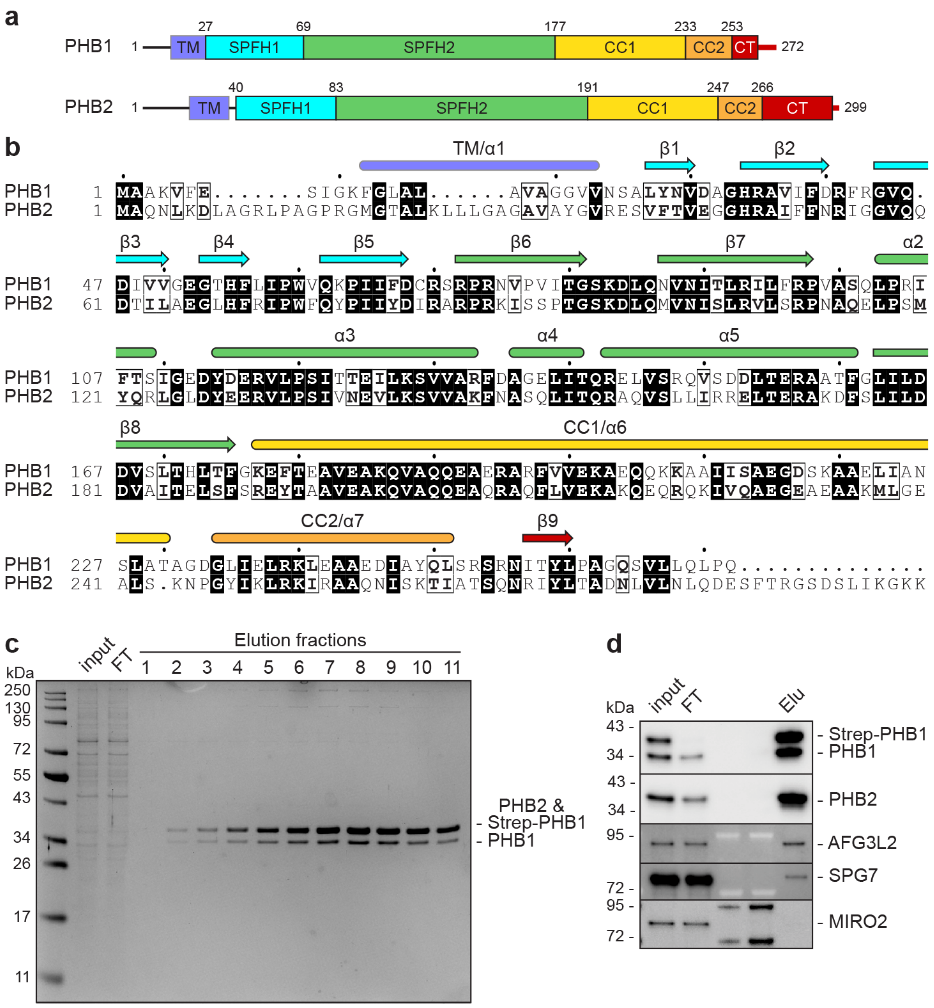

Supplementary Fig. 6. Alignment and domain organization of PHB1 and PHB2. a, Domain scheme of PHB1 and PHB2. **b,** Sequence alignment of human PHB1 and PHB2, with structural features noted above, colored according to domain. **c,** Coomassie stain of the PHB1/2 complex purified via N-terminally Strep-tagged PHB1. FT, flow-through. **d,** Immunoblotting of samples as in c for the indicated mitochondrial proteins. Elu, peak elution fraction. Source data are provided as a Source Data file.

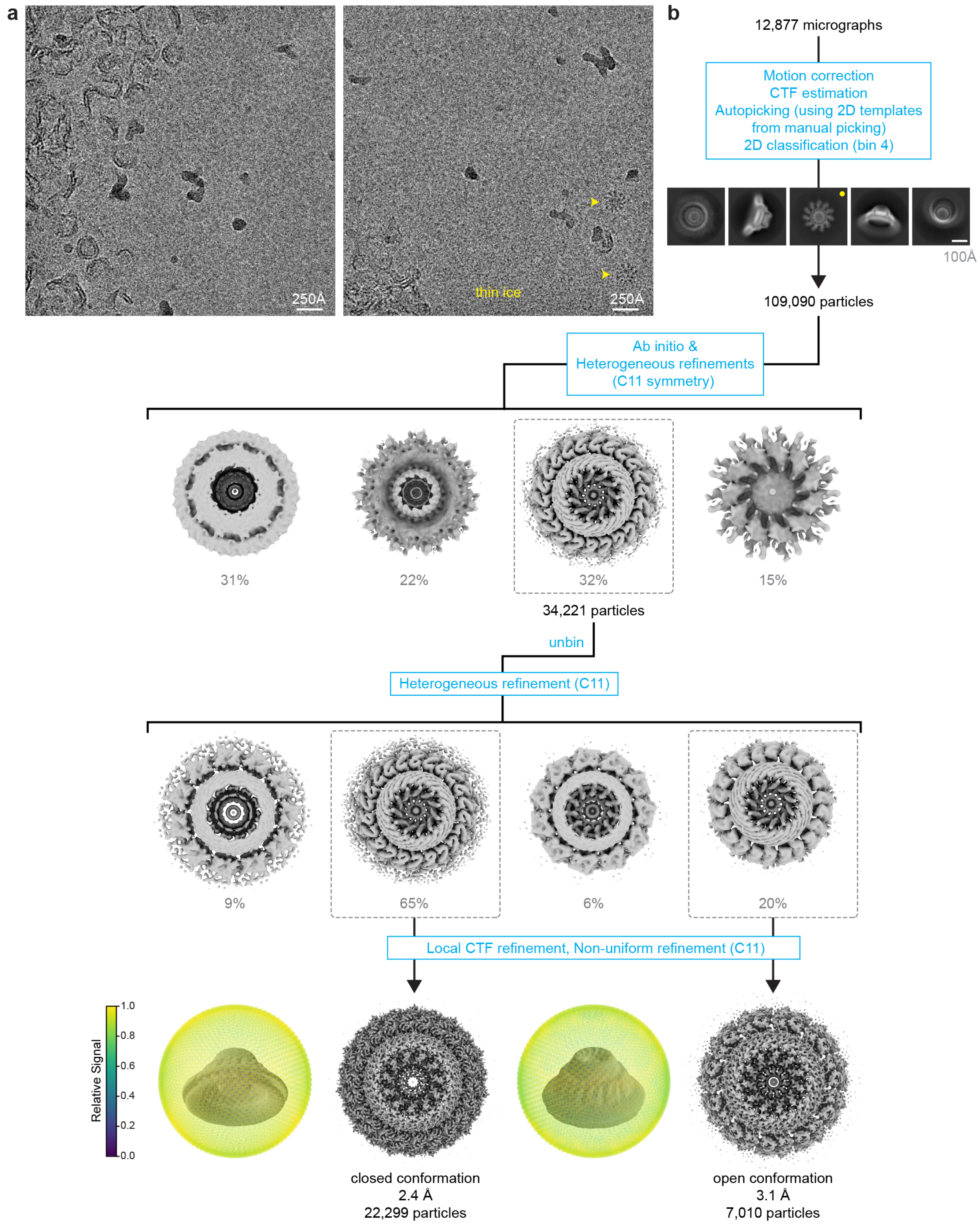

Supplementary Fig. 7. Cryo-EM data processing pipeline for the PHB1/2 complex. a, Representative micrographs of the PHB1/2 complex. **b,** Cryo-EM processing pipeline showing representative 2D classes, 3D volumes, and the angular distribution of particles to the left of each reconstruction. Note: 'Splayed out' particles (yellow arrows and dot) are solely in thin ice.

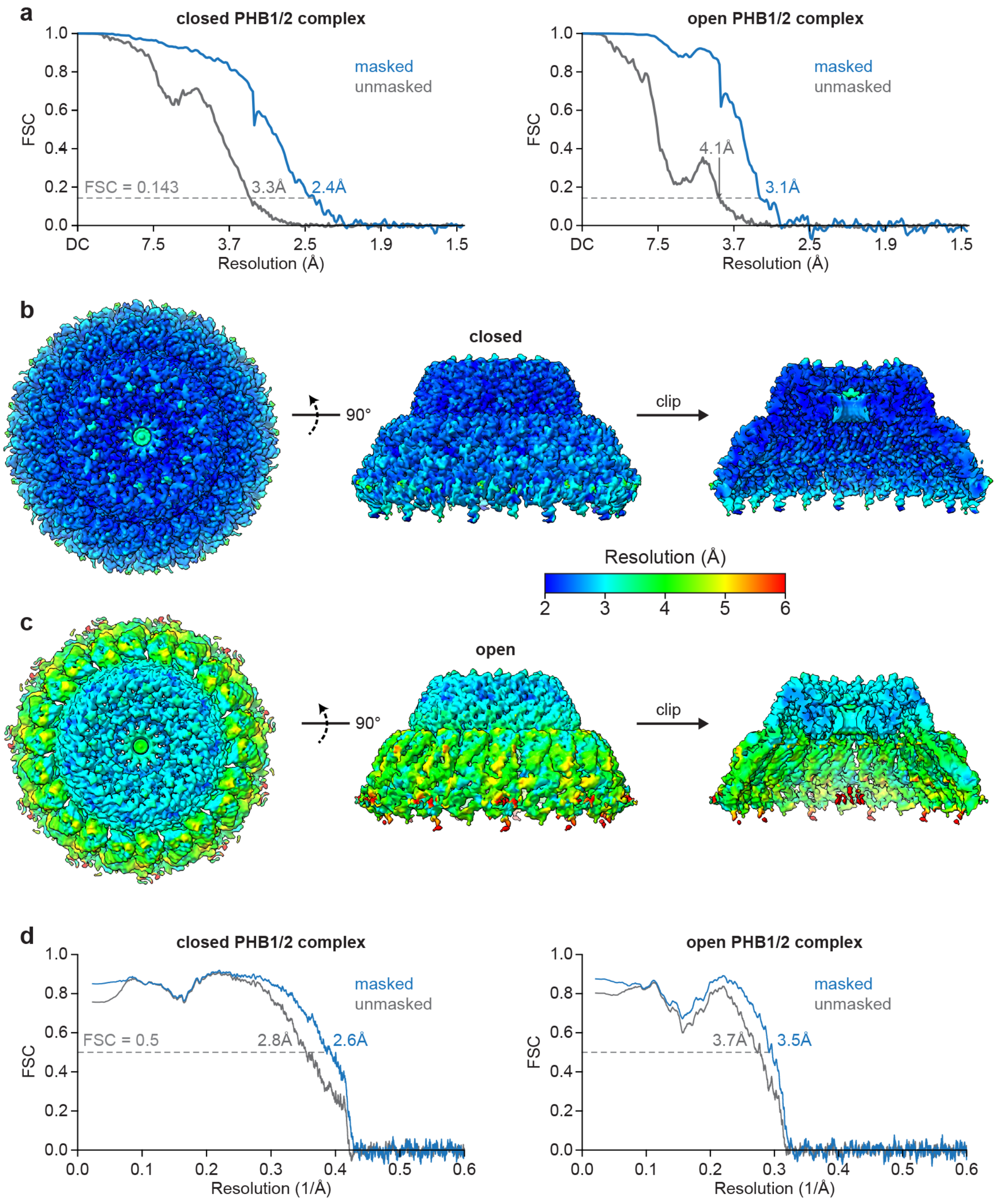

Supplementary Fig. 8. Quality of PHB1/2 maps and models. a, Fourier shell correlation (FSC) vs. resolution curves for the cryo-EM maps of the closed (left) or open (right) PHB1/2 complex. **b,c,** Top (left), side (middle), and clipped (right) views of the cryo-EM maps of the **b,** closed or **c,** open complex, colored by local resolution. **d,** Model vs. map FSC curves for the closed (left) or open (right) complex. Source data are provided as a Source Data file.

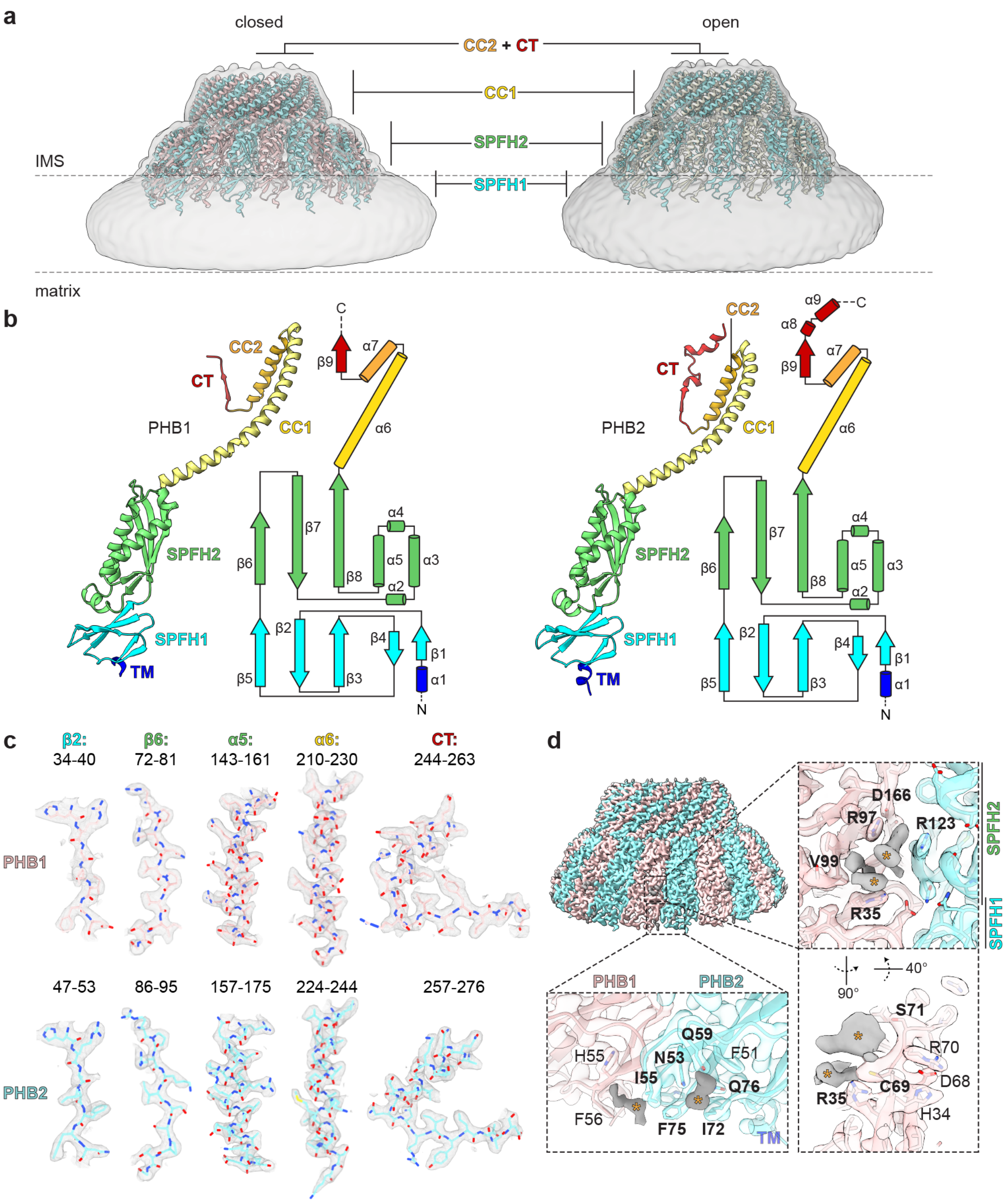

Supplementary Fig. 9. PHB1 and PHB2 domain organization and map-to-model fits. a, Model of the closed (left) or open (right) PHB1/2 complex superposed in low-pass filtered maps showing the detergent micelle as a proxy for the inner mitochondrial membrane (dashed lines) separating the intermembrane space (IMS) and the mitochondrial matrix. The placement of PHB1/2 domains are indicated. **b,** Model and secondary structural element organization of PHB1 (left) and PHB2 (right), colored by domain. **c,** Model fits for the indicated regions of PHB1 (top) or PHB2 (bottom) in the closed complex map, contoured at 5σ. **d,** Cryo-EM map of the PHB1/2 complex with insets showing additional unidentified densities (gray, orange asterisks) associated with the SPFH1 domains (bottom left) and at the SPFH1-SPFH2 interface between subunits (right) that may involve modification(s) of C69 of PHB1.

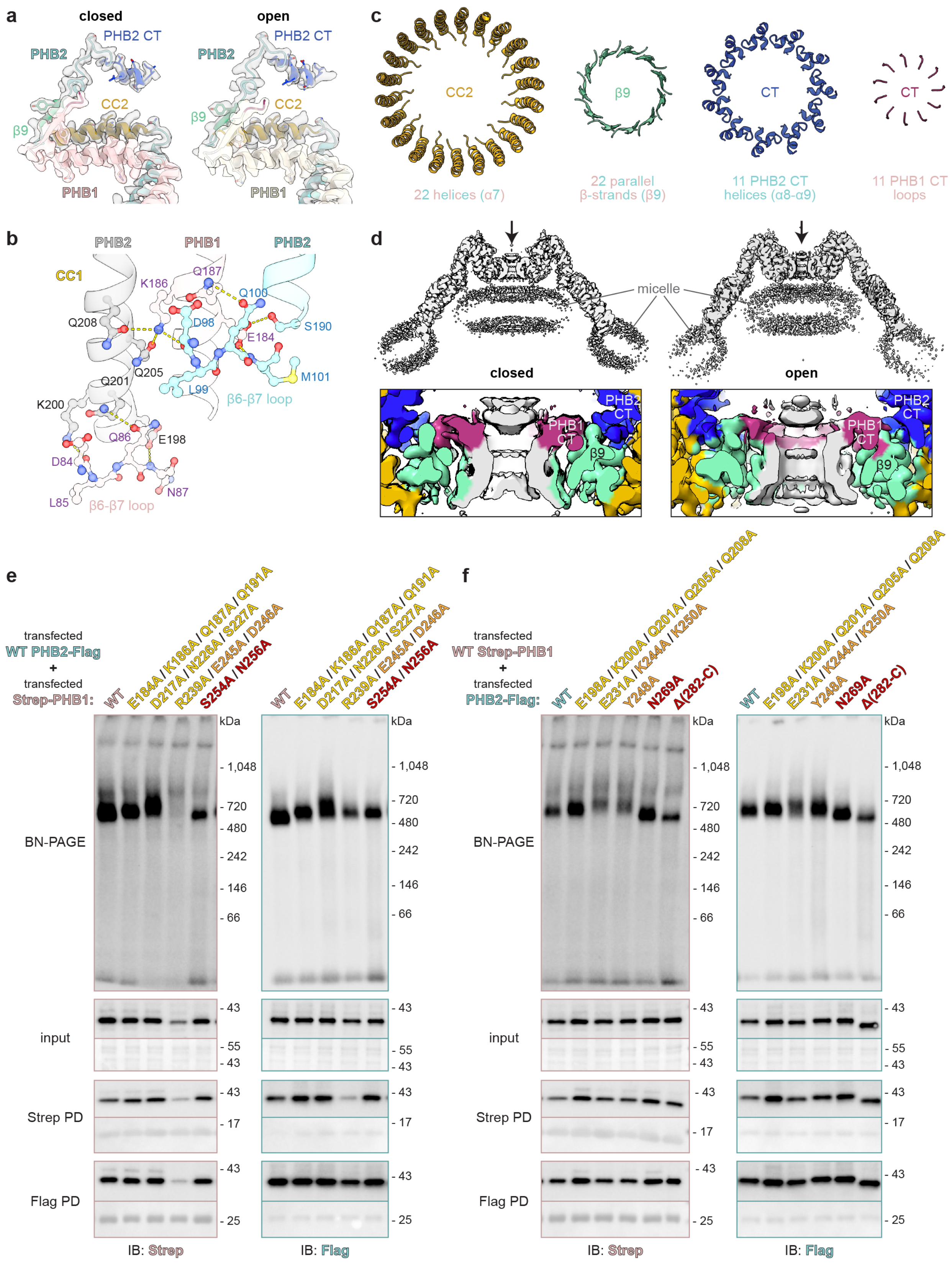
a
closed
open
PHB2 CT
PHB2
CC2
β9
PHB1
c
CC2
22 helices (α7)
β9
22 parallel
β-strands (β9)
CT
11 PHB2 CT
helices (α8-α9)
CT
11 PHB1 CT
loops
b
PHB2
PHB1
PHB2
CC1
K186
Q187
Q100
Q208
D98
S190
E184
M101
Q205
Q201
L99
β6-β7 loop
K200
Q86
E198
D84
N87
L85
β6-β7 loop
d
micelle
closed
open
PHB2 CT
PHB1 CT
β9
e
transfected
WT PHB2-Flag
+
transfected
Strep-PHB1:
WT
E184A / K186A / Q187A / Q191A
D217A / N226A / S227A
R239A / E245A / D246A
S254A / N256A
kDa
1,048
720
480
242
146
66
BN-PAGE
input
Strep PD
Flag PD
43
55
17
25
IB: Strep
IB: Flag
f
transfected
WT Strep-PHB1
+
transfected
PHB2-Flag:
WT
E198A / K200A / Q201A / Q205A / Q208A
E231A / K244A / K250A
Y248A
N269A
Δ(282-C)

Supplementary Fig. 10. Structural characteristics of the PHB1/2 complex. a, Map-to-model fit of C-terminal PHB1 and PHB2 elements in the closed and open conformations. Note: PHB2 contains a C-terminal extension (dark blue) not present in PHB1. **b,** Close-up view of the hydrogen bonding network between the N-terminal portion of the CC1 helix and the β6-β7 loop in the SPFH2 domain of an adjacent subunit. **c,** Isolated elements contributing to the four layers at the narrow end of the PHB1/2 complex cage, colored as in Fig. 6f. **d,** Side view of the cryo-EM map of the closed or open PHB1/2 complex, clipped near the center (top) shows a central channel (arrow) flanked by the PHB1 CT and the β9 ring (insets at bottom). **e,f,** BN-PAGE (top) or SDS-PAGE (bottom) and immunoblotting for N-terminally Strep-tagged PHB1 (left panels) or C-terminally Flag-tagged PHB2 (right panels) in HEK293T cells transfected to express equal levels of **e,** WT PHB2-Flag and the indicated Strep-PHB1 variants, or **f,** WT Strep-PHB1 and the indicated PHB2-Flag variants. SDS-PAGE and immunoblotting was performed before or after Strep or Flag pulldowns (PD) as indicated. CC1 mutations are indicated in yellow, CC2 mutations are indicated in orange, and CT mutations are indicated in red. Note: mutations in the CC2 helix are more likely to disrupt complex assembly and protein stability. Source data are provided as a Source Data file.
