# Supplementary material for: Structures of human organellar SPFH protein complexes
Source: Nat Commun. 2025 Nov 17;16:10064. doi: 10.1038/s41467-025-65078-3 (PMC12624060; doi:10.1038/s41467-025-65078-3)
Supplement: Supplementary file 2 — Reporting Summary [file 41467_2025_65078_MOESM2_ESM.pdf]

nature portfolio

Corresponding author(s): Shao

Last updated by author(s): Sep 5, 2025

Reporting Summary

Nature Portfolio wishes to improve the reproducibility of the work that we publish. This form provides structure for consistency and transparency in reporting. For further information on Nature Portfolio policies, see our Editorial Policies and the Editorial Policy Checklist.

Statistics

For all statistical analyses, confirm that the following items are present in the figure legend, table legend, main text, or Methods section.

n/a	Confirmed	
☐	☒	The exact sample size (*n*) for each experimental group/condition, given as a discrete number and unit of measurement
☒	☐	A statement on whether measurements were taken from distinct samples or whether the same sample was measured repeatedly
☒	☐	The statistical test(s) used AND whether they are one- or two-sided<br>*Only common tests should be described solely by name; describe more complex techniques in the Methods section.*
☒	☐	A description of all covariates tested
☒	☐	A description of any assumptions or corrections, such as tests of normality and adjustment for multiple comparisons
☒	☐	A full description of the statistical parameters including central tendency (e.g. means) or other basic estimates (e.g. regression coefficient) AND variation (e.g. standard deviation) or associated estimates of uncertainty (e.g. confidence intervals)
☒	☐	For null hypothesis testing, the test statistic (e.g. F, t, r) with confidence intervals, effect sizes, degrees of freedom and P value noted<br>*Give P values as exact values whenever suitable.*
☒	☐	For Bayesian analysis, information on the choice of priors and Markov chain Monte Carlo settings
☒	☐	For hierarchical and complex designs, identification of the appropriate level for tests and full reporting of outcomes
☒	☐	Estimates of effect sizes (e.g. Cohen's d, Pearson's r), indicating how they were calculated

Our web collection on statistics for biologists contains articles on many of the points above.

Software and code

Policy information about availability of computer code

Data collection	SerialEM v4.0.5, EPU3.6
Data analysis	cryoSPARC v4.3.1, RELION-4.0.1, deepEMhancer 20220530_cu10, ModelAngelo v1.0.14, ChimeraX v1.8, Phenix v1.19.2, Coot v0.9.8, Clustal Omega (https://www.ebi.ac.uk/jdispatcher/msa/clustalo), ESPript 3.0 (https://espript.ibcp.fr/ESPript/ESPript/)

For manuscripts utilizing custom algorithms or software that are central to the research but not yet described in published literature, software must be made available to editors and reviewers. We strongly encourage code deposition in a community repository (e.g. GitHub). See the Nature Portfolio guidelines for submitting code & software for further information.

Data

Policy information about availability of data

All manuscripts must include a data availability statement. This statement should provide the following information, where applicable:

- Accession codes, unique identifiers, or web links for publicly available datasets
- A description of any restrictions on data availability
- For clinical datasets or third party data, please ensure that the statement adheres to our policy

The cryo-EM maps generated in this study have been deposited in the Electron Microscopy Data Bank (EMDB) under accession codes EMD-70263 [https://www.ebi.ac.uk/emdb/EMD-70263] (the Erlin1/2 complex), EMD-70267 [https://www.ebi.ac.uk/emdb/EMD-70267] (the closed PHB1/2 complex), and EMD-70268 [https://www.ebi.ac.uk/emdb/EMD-70268] (the open PHB1/2 complex). The atomic coordinates have been deposited in the Protein Data Bank (PDB) under accession codes PDB 9O9U [https://doi.org/10.2210/pdb9O9U/pdb] (the Erlin1/2 complex), PDB 9O9Z [https://doi.org/10.2210/pdb9O9Z/pdb] (the closed PHB1/2

complex), and PDB 9OA0 [https://doi.org/10.2210/pdb9OA0/pdb] (the open PHB1/2 complex). All other data are available within the article and its Supplementary Information. Source data are provided with this paper. Correspondence and requests for materials should be directed to S.S.

Research involving human participants, their data, or biological material

Policy information about studies with human participants or human data. See also policy information about sex, gender (identity/presentation), and sexual orientation and race, ethnicity and racism.

Reporting on sex and gender	N/A
Reporting on race, ethnicity, or other socially relevant groupings	N/A
Population characteristics	N/A
Recruitment	N/A
Ethics oversight	N/A

Note that full information on the approval of the study protocol must also be provided in the manuscript.

Field-specific reporting

Please select the one below that is the best fit for your research. If you are not sure, read the appropriate sections before making your selection.

[x] Life sciences [ ] Behavioural & social sciences [ ] Ecological, evolutionary & environmental sciences

For a reference copy of the document with all sections, see nature.com/documents/nr-reporting-summary-flat.pdf

Life sciences study design

All studies must disclose on these points even when the disclosure is negative.

Sample size	No statistical methods involving predetermined sample sizes were used. The size of cryo-EM samples were established by semi-automated particle-picking algorithms and further classified by established cryo-EM data processing alogrithms in cryoSPARC or RELION. No other statistical tests were performed.
Data exclusions	Established single particle image processing algorithms may exclude or weight particles. No other data were excluded from analyses.
Replication	All protein complexes analyzed by cryo-EM were purified at least twice independently with reproducible results. All biochemical assays were repeated at least three independent times with reproducible results.
Randomization	Established single particle imaging processing algorithms randomly split cryo-EM particle images into two halves during refinement. Randomization is not required for other experiments in the study because all independent variables in biochemical experiments were directly controlled, and all variant comparisons were subjected to the same experimental conditions.
Blinding	Blinding is not relevant to our study because no statistical tests were performed on comparisons and all biochemical experiments were directly controlled.

Reporting for specific materials, systems and methods

We require information from authors about some types of materials, experimental systems and methods used in many studies. Here, indicate whether each material, system or method listed is relevant to your study. If you are not sure if a list item applies to your research, read the appropriate section before selecting a response.

Materials & experimental systems

n/a	Involved in the study	
[ ]	[x]	Antibodies
[ ]	[x]	Eukaryotic cell lines
[x]	[ ]	Palaeontology and archaeology
[x]	[ ]	Animals and other organisms
[x]	[ ]	Clinical data
[x]	[ ]	Dual use research of concern
[x]	[ ]	Plants

Methods

n/a	Involved in the study	
[x]	[ ]	ChIP-seq
[x]	[ ]	Flow cytometry
[x]	[ ]	MRI-based neuroimaging

Antibodies

Antibodies used	The following antibodies were used for immunoblotting: HRP-conjugated anti-FLAG M2 (Sigma A8592, 1:5,000), HRP-conjugated StrepTactin (Bio-rad 1610381, 1:5,000), anti-Strep II (Abcam ab76949), anti-Erlin1 (Invitrogen PA5-19152, 1;1,000), anti-Erlin2 (Abcam ab128924, 1:4,000), anti-PHB1 (Invitrogen PA5-27329, 1:1,000), anti-PHB2 (Invitrogen PA5-14133, 1:1,000), anti-AFG3L2 (Abcam ab68023, 1:500), anti-SPG7 (Novus NBP2-01860, 1:500), and anti-MIRO2 (Abcam ab224089, 1:1,000) were used for immunoblotting, together with HRP-conjugated goat anti-rabbit IgG (Jackson ImmunoResearch 111-035-003, 1:5,000), goat anti-mouse IgG (Jackson ImmunoResearch 115-035-003, 1:5,000), and rabbit anti-goat IgG (R&D Systems HAF017, 1:1,000)
Validation	Antibodies were all validated by immunoblotting by the manufacturer of each antibody. HRP-conjugated anti-FLAG M2 (https://www.sigmaaldrich.com/US/en/product/sigma/a8592), HRP-conjugated StrepTactin (https://www.bio-rad.com/en-us/sku/1610381-precision-protein-streptactin-hrp-conjugate-125-ul?ID=1610381), anti-Strep II (https://www.abcam.com/en-us/products/primary-antibodies/strep-tag-ii-antibody-ab76949), anti-Erlin1 (https://www.thermofisher.com/antibody/product/ERLIN1-Antibody-Polyclonal/PA5-19152), anti-Erlin2 (https://www.abcam.com/en-us/products/primary-antibodies/erlin-2-antibody-epr8089-ab128924), anti-PHB1 (https://www.thermofisher.com/antibody/product/Prohibitin-Antibody-Polyclonal/PA5-27329), anti-PHB2 (https://www.thermofisher.com/antibody/product/REA-Antibody-Polyclonal/PA5-14133), anti-AFG3L2 (https://www.abcam.com/en-us/products/primary-antibodies/afg3l2-antibody-ab68023), anti-SPG7 (https://www.novusbio.com/search?keywords=Novus+NBP2-01860), anti-MIRO2 (https://www.abcam.com/en-us/products/primary-antibodies/miro2-antibody-ab224089), HRP-conjugated goat anti-rabbit IgG (https://www.jacksonimmuno.com/catalog/products/111-035-003), goat anti-mouse IgG (https://www.jacksonimmuno.com/catalog/products/115-035-003), rabbit anti-goat IgG (https://www.rndsystems.com/products/goat-igg-hrp-conjugated-antibody_haf017).Erlin1, Erlin2 and PHB1 antibodies were additionally validated for specificity in our pulldowns of recombinantly expressed Strep- and Flag-tagged proteins. Strep II and Flag antibodies were additionally verified for their specificity in lysates expressing tagged proteins.

Eukaryotic cell lines

Policy information about cell lines and Sex and Gender in Research

Cell line source(s)	Expi293F (A14527) and Flp-In 293 T-REx (R78007) cells were originally from ThermoFisher Scientific. HEK293T cells (CRL-3216) were originally from ATCC. Expi293F cells stably expressing N-terminally Strep-tagged PHB1 was generated using standard lentiviral production from HEK293T cells and transduction into Expi293F cells, followed by selection, as described in the Methods section. Erlin1 knockout, Erlin2 knockout and Erlin1 and Erlin2 double-knockout cell lines were made using CRISPR-Cas9 technologies using specific gRNAs as described in the Methods section.
Authentication	The Expi293F cell line stably expressing N-terminally Strep-tagged PHB1, Erlin1 KO, Erlin2 KO and Erlin1/2 DKO cell lines were validated by Western blotting. No other validations were performed.
Mycoplasma contamination	Cell lines were not tested for mycoplasma contamination.
Commonly misidentified lines (See ICLAC register)	No commonly misidentified cell line was used in the study.

Plants

Seed stocks	N/A
Novel plant genotypes	N/A
Authentication	N/A
